# Identification of a core set of Campylobacter jejuni flagella modification genes and a reversible non-motile maf3 phenotype

**DOI:** 10.1099/mic.0.001698

**Published:** 2026-04-28

**Authors:** Lickson Munjoma, Christopher D. Bayliss

**Affiliations:** 1 Division of Microbiology and Infection, School of Biological and Biomedical Sciences, College of Life Sciences, University of Leicester, Leicester, UK

**Keywords:** *Campylobacter*, genomics, motility, phase variation

## Abstract

Assembly of flagella filaments in *Campylobacter jejuni* requires post-translational O-glycosylation of the flagellin proteins with pseudaminic acid, legionaminic acid and related derivatives. This species is unusual as the addition of pseudaminic acid replaces the requirements for toll-like receptor 5 motifs present in most bacterial flagellin proteins. The distribution and functions of the motility accessory factor genes, encoding glycosyltransferases, responsible for the modification of *C. jejuni* flagellin across this species, are not fully understood. Disruption of these genes can affect autoaggregation, motility, colonization and biofilm formation. Bioinformatic analyses for the presence/absence of biosynthetic and transferase genes were performed across 16,130 *C*. *jejuni* genomes for homologues of genes contained in the *C. jejuni* strain NCTC11168 flagella locus. The presence of four putative transferases – *cj1295*, *maf7*, *cj1305* and *cj1306* – correlated with pseudaminic acid biosynthetic genes, whereas legionaminic acid biosynthetic genes were correlated with another five *maf* genes. A full NCTC11168-like *maf* gene set was present in 42.7% of isolates, with three genes, *maf3*, *maf4* and *maf5*, being present in >80% of isolates. Mutagenesis of *maf3* in *C. jejuni* strain NCTC11168 resulted in a reversible, non-motile phenotype. Motile revertants of this mutant had *maf4* and *maf7* in the ON phase variation state and/or possessed a single-nucleotide alteration in *maf5*. These findings are indicative of specific *maf* genes being responsible for the addition or modification of major flagella glycans in large groups of *C. jejuni* strains and of redundant or compensatory gene functions.

## Data Access

All data are either included within the manuscript or are openly available at the University of Leicester Research Repository, accessible via Bayliss, Christopher; Munjoma, Lickson (2025). Genomic and experimental analysis of motility genes of *Campylobacter jejuni*, University of Leicester, Dataset, https://doi.org/10.25392/leicester.data.29681483.v1. Assembled genome sequence data for the 16,130 are available in the *Campylobacter jejuni*/*coli* PubMLST database available at https://pubmlst.org/organisms/campylobacter-jejunicoli. Genome sequence data for the *maf3* mutant is available at accession number JBPZXT000000000 in National Center for Biotechnology Information (NCBI) GenBank. Supplementary materials available on Figshare at https://doi.org/10.6084/m9.figshare.31411913 [1].

## Introduction

Campylobacteriosis is a highly prevalent and widely occurring disease caused by a range of *Campylobacter* species, of which *Campylobacter jejuni* is the most prevalent [2]. The major sources of *C. jejuni* infections are contaminated meat products, predominantly poultry, and environmental sources. *C. jejuni* lineages show varying degrees of specificity, with some lineages described as poultry-specific and others as generalists [3]. Campylobacters exhibit flagella-based motility, with motility and the flagella being major determinants of human and animal infections.

**Fig. 1.**
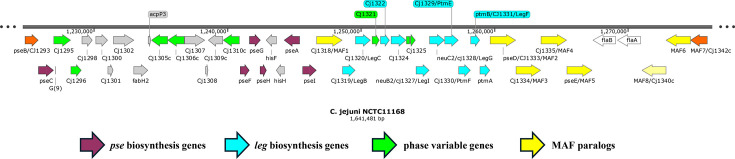
Cluster of flagella structural, biosynthetic and modification genes in the genome sequence of *C. jejuni* strain NCTC11168. This diagram depicts the genes from *cj1293* to *cj1342* (marked in orange) that constitute the flagella locus. Genes are shown as arrowed rectangles. The genes encoding the flagellin structural genes, *flaA* and *flaB*, are in white. The pseudaminic (pse) and legionaminic (leg) acid biosynthetic genes are in magenta and light blue, respectively. Genes in yellow are the motility accessory genes (*maf*) as identified by Karlyshev *et al*. (2005). Genes in green are subject to phase variation due to polyC/G tracts (note that *maf1*, *maf4* and cj1342 are also subject to PV). Genes of unknown function are grey. The image was rendered in SnapGene version 8.2.1.

The flagella of *C. jejuni* are required for motility, colonization, biofilm formation and autoaggregation [4,5]. These polar-situated organelles are comprised of polymerized flagellin proteins that are extensively decorated with nonulosonic acids [6]. The primary carbohydrate of importance for flagella synthesis is pseudaminic acid, with the work of Goon *et al*. [7] indicating that this modification is required for flagella assembly in *C. jejuni*. Some, but not all, *C. jejuni* strains also exhibit modification of the flagella with legionaminic acid, and both glycans can be further modified with acetyl, acetamidino and glutamic acid modifications [6,8, 9]. Large sets of biosynthesis and glycosyltransferase genes are responsible for the post-translational modification of flagellin proteins with these glycans. The motility accessory factor genes (*maf*) were detected due to their effects on motility and vary in number between strains [10]. Karlyshev *et al*. [10] identified seven paralogous *maf* genes within the *C. jejuni* strain NCTC11168 genome and also showed that a *maf5* deletion mutation led to a reversible, non-motile phenotype. The flagella biosynthetic genes, *maf* genes and several genes of unknown function are present with the two flagellin-encoding genes, *flaA* and *flaB*, in a large region of the *C. jejuni* genome (Fig. 1 [11]). Variations in gene content and sequence are presumed to determine variations in the structural modification and function of *C. jejuni* flagella between strains and within clonal populations.

The functions of the *maf* genes have been explored in three *C. jejuni* strains: 81–176, 108 and 480. Mutations in the *maf2*, *maf3*, *maf6* and *maf7* genes did not affect the motility of *C. jejuni* strain 81-176, although a *maf2* (*pseD*) mutant exhibited reduced autoaggregation, with this gene being involved in attachment of the acetamidino form of pseudaminic acid [12]. Golden *et al*. [13] showed that *maf1* and *maf2* mutants in *C. jejuni* strain 480, a clinical isolate, caused 36% and 14% decreases, respectively, in motility after 18 h. Mutations in *maf4* were found to decrease motility and delay autoaggregation in *C. jejuni* strain 108, with this phenotype being linked with the addition of acidic functional groups to pseudaminic acid [14]. Subsequently, Meng *et al*. [15] found that a *maf4* deletion inhibited legionaminic acid-dependent flagellin glycosylation in this strain, suggestive of dual catalytic functions of glycotransferases in the presence of pseudaminic acid and legionaminic acid.

Another characteristic feature of *C. jejuni* is phase variation (PV) mediated by polyC or polyG repeats present in the reading frame or promoters of multiple genes [11]. These repeat tracts are hypermutable, resulting in reversible, high-frequency generation of ON and OFF variants [16]. The *maf1*, *maf4* and *maf7* genes are all subject to translational PV [10]. Several other genes known or putatively involved in flagella modification, including *cj1295* and *cj1296*, are also subject to PV [17]. The contributions of PV of these genes to flagella glycosylation and functions are poorly understood.

In order to understand whether the *maf* genes are essential for the addition of pseudaminic or legionaminic acid to *C. jejuni* flagella, we investigated the distribution of the *maf* and other co-localized genes of unknown function across multiple *C. jejuni* isolates. This bioinformatic analysis revealed that *maf3* and *maf5* were part of a core NCTC11168-like *maf* gene set present in most *C. jejuni* isolates and led to the construction of a *maf3* knockout mutant in the NCTC11168 strain. This mutant had a non-motile phenotype that was subject to reversion by switching ON of the *maf4* and *maf7* genes or mutations in *maf5*. Our findings provide new insights into the importance of *maf* genes to *C. jejuni* flagella glycosylation and evidence of compensatory flagella glycan modification pathways.

## Material and methods

### Bioinformatic analyses of *C. jejuni* genome sequences

Nucleotide sequences of specific genes were obtained from the genome sequence of *C. jejuni* strain NCTC11168 (NC_002163). These sequences were used to query the *Campylobacter* PubMLST database (https://pubmlst.org/) against 16,130 genome sequences of *C. jejuni* isolates [18]. Isolates were considered to have the gene sequence present (positive) under the following conditions: percentage identity, ≥80%; coverage, ≥50%. Using RStudio, the total number of positive isolates was expressed as a proportion of the total population size for each respective clonal complex and plotted as a heat map. For *maf4*, 200 bp of flanking sequence from either end of the gene was included in order to obtain a match that is between *maf3* and *maf5* as found in the strain NCTC11168 genome. The *maf4* hits were also assessed for synteny with these two flanking genes.

### Bacterial strains and growth conditions

A chicken-adapted variant of *C. jejuni* strain NCTC11168 (*C. jejuni* strain NCTC11168ca), provided by Professor Julian Ketley and Dr Richard Haigh, was utilized for all experiments with this strain. *C. jejuni ΔflaA* was produced by Dr Khaloud Alarjani [19]. Other *C. jejuni* strains were generated during this study (Table 1). All *C. jejuni* strains were grown on blood agar (Oxoid, Basingstoke, UK) supplemented with 5% horse blood or in Mueller–Hinton broth (Oxoid) at 42 °C under microaerophilic conditions of 85% N_2_, 10% CO_2_ and 5% O_2_ in a variable atmosphere incubator (VAIN) cabinet (Don Whitley Scientific Ltd., Shipley, UK). Broth cultures were grown with shaking at 50 r.p.m. (Vibrax VXR, IKA, Germany). Media were supplemented with 10 µg ml^−1^ vancomycin and 5 µg ml^−1^ trimethoprim (Sigma) as standard antibiotics and 50 µg ml^−1^ kanamycin (Sigma) or 10 µg ml^−1^ chloramphenicol during mutant selection. Strains and mutants were cryopreserved in 25% (v/v) glycerol at −80 °C. Inoculum populations were prepared by plating glycerol stocks onto agar plates, incubating for 2 days, sub-culturing by swabbing a single colony or mixed population onto fresh agar plates and repeating the overnight incubation to patches for bacterial suspensions.

**Table 1.** Bacterial strains and mutants

Bacterial strain	Description	Source
*C. jejuni* NCTC1168ca	Chicken-adapted hypermotile derivative of *C. jejuni* strain NCTC 11168	Haigh and Ketley, unpublished data
*C. jejuni* NCTC1168ca *ΔflaA* Ca	NCTC 11168 Δ*flaA*::kan/natural transformation	Alarjani and Bayliss, unpublished data
*C. jejuni* NCTC1168ca *Δmaf3::kan^R^*	Deletion of the *maf3* gene by insertion of *kan^R^*	This study
*C. jejuni* NCTC1168ca Δ*maf3*::*maf3*	Complementation of Δ*maf*3 deletion by insertion of the *maf3* coding sequence into the *cj0046* locus under control of the *metK* promoter	This study

### Mutant construction

The *maf3* flanking regions were amplified with a Q5 PCR kit using *C. jejuni* NCTC11168ca chromosomal DNA as template and relevant primer pairs (Table S1, available in the online Supplementary Material). The reverse primers had 5′ regions complementary to a kanamycin cassette. The complementary regions facilitated ligation of a resistance marker between the homology arms during HiFi assembly of the three fragments into a single fragment. This combined product (referred to as MAF3_long_frag) was amplified with the MAF3_L_Arm_F and MAF3_R_Arm_R primers using the Q5 PCR kit and the HiFi product as template. The MAF3_long_frag product was cloned into a pGEM T-easy vector. The pGEM::Δ*maf*3 construct was used for natural transformation of *C. jejuni* NCTC11168ca. Transformants were selected on kanamycin and confirmed by PCR with the MAF3_L_Arm_F and MAF3_R_Arm_R primers.

To complement the *Δmaf3* mutant, the full-length *maf3* gene was cloned into the pcmetK vector (provided by Dr Molly Webster), which contains a chloramphenicol cassette, a BsmBI site for linearization, a metK promoter and homology arms compatible with the pseudogene, *cj0046*. The *maf3* gene was amplified with a Q5 PCR kit using *C. jejuni* NCTC11168ca chromosomal DNA as template and Metk_MAF3_F and Metk_MAF3_R primers. These primers had 5′ regions complementary to the BsmBI site, enabling insertion into pcmetK. Ligation into pcmetK used the HiFi method and was verified by PCR with CJ0046_iLOV_F and Cat_promoter_R primers. pcmetK::MAF3 was used for natural transformation of the Δ*maf3* mutant. Transformants were selected on chloramphenicol and confirmed by PCR with the CJ0046_iLOV_F and Cat_promoter_R primers.

### Motility assays

Bacterial cultures were incubated overnight on Mueller Hinton Agar (MHA) plates and adjusted to an OD_600nm_ of 0.3. Assays were performed with 0.4% (w/v) MHA plates by inoculation of 10 µl of a bacterial suspension into the centre of the plate. Plates were incubated under microaerobic conditions in an elevated region of the VAIN cabinet to avoid condensation. Halo diameter (mm) was measured, and images were obtained across 96 h.

### GeneScan analysis of the repeat tracts of phase-variable genes

Phase-variable genes within the flagella glycosylation locus were analysed for changes in tract length as described by Lango-Scholey *et al*. [20]. Briefly, samples consisting of sweeps of multiple colonies or single colonies from inocula were resuspended in dH_2_O, denatured by heating to 95 °C for 5 min and used as DNA templates for PCR. Genomic DNA purified from *C. jejuni* strain NCTC11168ca was used as the control for GeneScan analysis. The repeat numbers for all PV genes of this DNA preparation were characterized by dideoxy Sanger sequencing prior to use in PV analyses. Fluorescently labelled primers were used in multiplex PCR reactions and A-tailing reactions. PCR aliquots of 2 µl were mixed with 8.75 µl formamide and 0.25 µl GS600LIZ size standard and then subjected to analysis on an ABI 3500xl autosequencer. Autosequencer files were analysed with psanalyse to determine repeat numbers and expression states relative to the control DNA.

### Whole-genome sequencing

Whole-genome sequencing was performed by MicrobesNG genome sequencing service (https://microbesng.com). Purified genomic DNA was resuspended in 10 mM Tris-HCl, pH 8.5, to a concentration of 30 ng µl^−1^ in aliquots.

### Statistical analysis

Statistical analyses and graphs were performed in RStudio, R version 4.4.1 (2024-06-14), ‘Race for Your Life’ [21]. Shapiro–Wilk tests were used to test for normality, and data were normalized by the bestNormalize package (version 1.9.1) if *P*<0.05.

## Results

### Differential clustering of *maf* genes with *pse* and *leg* biosynthetic genes

**Fig. 2.**
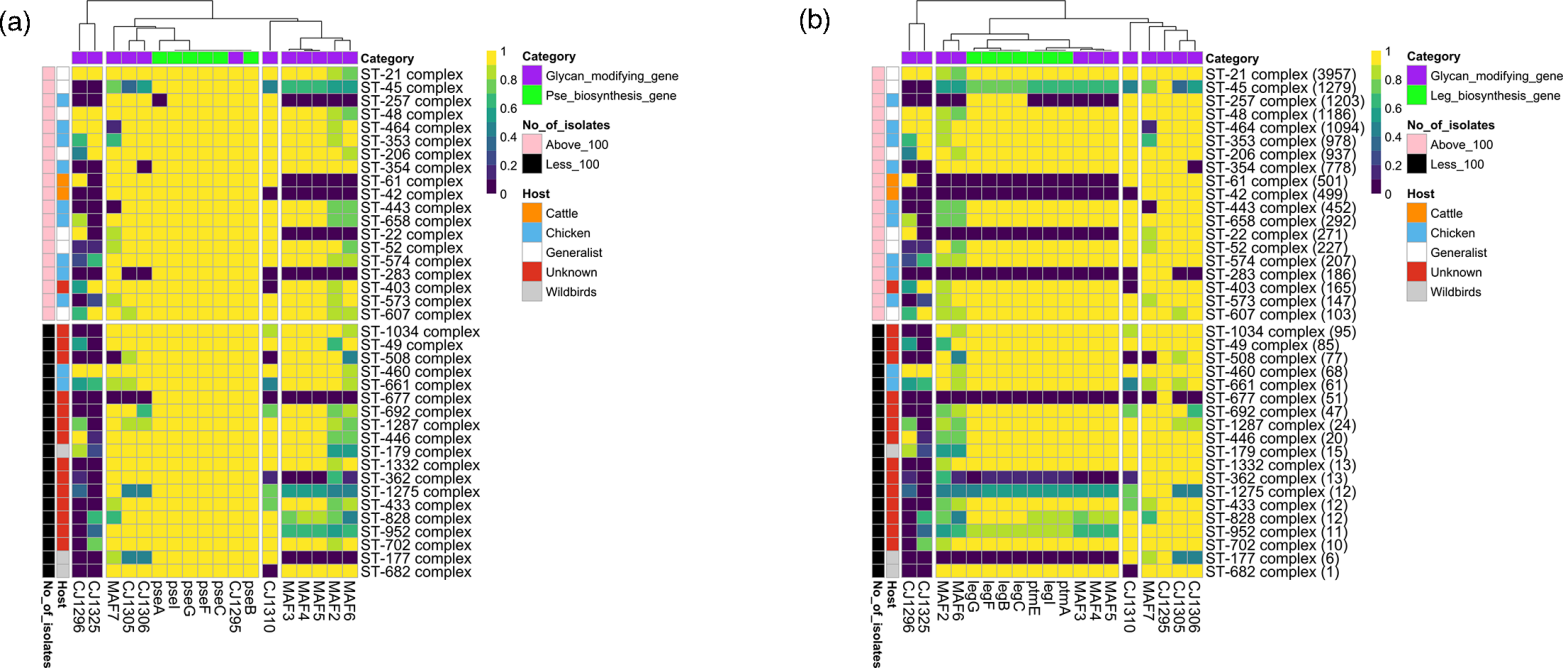
Associations between glycan biosynthetic and modifying genes across multiple *C. jejuni* clonal complexes. The sequences of 25 flagella locus genes from *C. jejuni* strain NCTC11168 were used as query sequences against 16,130 *C. jejuni* isolates in the *Campylobacter* PubMLST database. Isolates with the gene present (identity ≥80%; coverage ≥50%) were counted and expressed as a proportion of the total number of isolates in each clonal complex. The presence/absence proportions for each gene associated with either pseudaminic acid (a) or legionaminic acid (b) biosynthesis and flagella-modifying genes were plotted as a heat map, which was then subject to hierarchical clustering. The number of isolates per clonal complex is in parentheses (see b), and these values were used to sort the rows into descending order of coverage for both panels. An arbitrary cut-off of 100 isolates per clonal complex was used to split the heatmaps into major (top panel) and minor (bottom panel) complexes. Clonal complexes were annotated for host specialism as reported by Sheppard *et al*. and Mourkas *et al.* [22,38]. A set of 1,035 isolates was not assigned to any ST complex and was excluded from this analysis. Purple to yellow represents population proportions from 0 (0%) to 1 (100%), respectively.

To examine the conservation of the *maf* genes across *C. jejuni* strains and to identify associations of these genes with the major glycans, a bioinformatic analysis of the presence or absence of the biosynthetic and known/putative transferase genes for these glycans was performed using 16,130 *C*. *jejuni* genome sequences within the *Campylobacter* PubMLST database (Data File S1). The searches encompassed 12 biosynthetic genes, 6 *maf* genes and 6 other genes of unknown function that are all present in the flagella locus of *C. jejuni* strain NCTC11168 (Fig. 1). Identity and coverage scores were very high for genes encoding the biosynthetic loci but more variable for the other genes in this locus (Table 2). Gene presence was based on arbitrary values of ≥80% identity and ≥50% coverage. These data were utilized to assess the prevalence of each gene in 38 clonal complexes to determine whether lineages differed in flagella glycosylation capabilities (Fig. 2, Data File S1). The *maf1* and *maf4* genes are identical in the NCTC11168 genome sequence. For *maf4*, 200 bp of each flanking sequence was included in the blast search, and matches were compared for synteny with *maf3* and *maf5*, as these genes are all co-located in the NCTC11168 genome sequence (Fig. 1). Conversely, *maf1* was excluded due to issues with identifying syntenic matches. For high identity/coverage hits, *maf4* was located adjacent to one or both of these genes in 72.5% of the blast matches and in a distal location in only 0.5% of cases (indicative of a match to *maf1*). For 28% of isolates, all three genes were on separate contigs, possibly a result of poor chromosome assembly.

**Table 2.** Number of *C. jejuni* isolates with ≥80% identity and 50–100% coverage for selected flagella glycosylation genes

Gene	Gene length (bp)*	%Coverage of gene length				Total†	Percentage‡
		>99%	99–90%	90–80%	80–50%		
*pseA*	1137	14838	12	5	44	14899	92%
*legB*	972	12758	1269	5	13	14045	87%
*ptmA*	771	12777	11	9	7	12804	79%
*maf2*	1962	5252	526	588	4231	10597	66%
*maf3*	1860	12073	228	42	434	12777	79%
*maf4*§	2345	5385	2128	1052	2205	10040	62%
*maf5*	1887	11214	295	12	1189	12710	79%

*Gene length in strain NCTC11168.

†Total, number of isolates where gene is present as defined by ≥80% identity and ≥50% coverage.

‡Percentage, total divided by all isolates examined (*n*=16,130).

§Coverage for *maf4* is frequently reduced to 80% or less due to contig breaks occurring in the repeat tract.

Six genes involved in pseudaminic acid biosynthesis were highly present across all clonal complexes, with only *pseA* being absent in ST-257 (Fig. 2). In contrast, seven genes involved in legionaminic acid synthesis were completely absent from seven clonal complexes (ST: 177, 22, 283, 362, 42, 61, 677) or were absent in 10–40% of isolates of five other clonal complexes (ST: 1275, 257, 45, 828, 952). The rest of the genes clustered differentially according to each major glycan. Four genes – *cj1295*, *maf7*, *cj1305* and *cj1306* – clustered with pseudaminic acid biosynthetic genes (Fig. 2), with a correlation analysis indicating a very strong positive correlation with *cj1295* (Fig. S1). Similarly, six *maf* genes (*maf2–6*) clustered very strongly with the legionaminic acid biosynthetic genes (Figs 2 and S1). These associations are indicative of potential function links between subsets of flagella-modifying genes and legionaminic or pseudaminic acid biosynthesis genes.

### An equal distribution exists between isolates with both *maf* operons and isolates with a mixture of *maf* genes

**Fig. 3.**
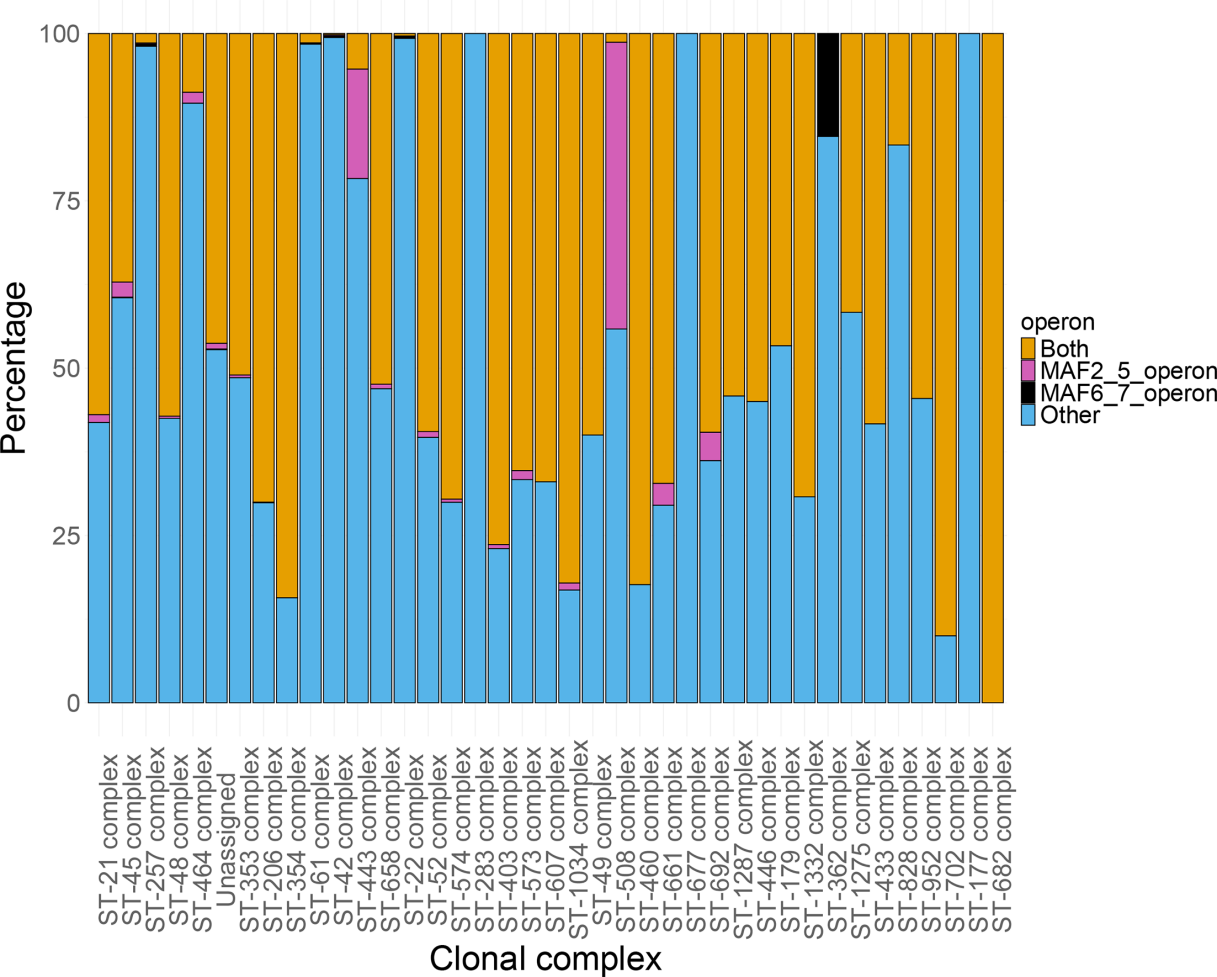
High prevalence of both *maf* operons across *Campylobacter* clonal complexes. The sequences of seven maf genes from *C. jejuni* strain NCTC11168 were used as query sequences against 16,130 *C. jejuni* isolates in the *Campylobacter* PubMLST database, and the presence/absence of each gene was determined (see Fig. 2). Isolates were then assessed for the presence of the four genes of the *maf2_5* operon or the three genes of the *maf6_8* operon. Isolates were assigned as having an intact *maf2_5* or *maf6_8* operon when all the genes (*n*=7) of the respective operon were present. Isolates were then categorized as having both operons intact (both), only one operon intact (*maf2_5*, only four MAF genes; *maf6_8*, only three MAF genes) or partial versions of one or both operons (other, with a MAF gene number of ≤6). The total number of isolates for each operon group was expressed as a percentage of the total number of isolates in each clonal complex. The number of isolates per clonal complex ranged from 1 to 3,957, while 1,035 isolates were not assigned to any ST complex (unassigned group). ST complexes are ordered in descending order of the number of isolates per complex from left to right.

Six of the seven *maf* genes found in *C. jejuni* strain NCTC11168 are located in two operons (Fig. 1). The maf2_5 operon consists of four genes, *maf2*, *maf3*, *maf4* and *maf5*, located downstream of the *flaAB* operon, and the maf6_7 operon, which includes three genes, *cj1340* (referred to as *maf8* herein), *maf6* and *maf7*, and is located upstream of the *flaAB* operon. The presence and composition of these two operons were determined by performing blast searches across 16,130 isolates and 38 clonal complexes of *C. jejuni* with sequences from *C. jejuni* strain NCTC11168. Analysis revealed that 42.7% (6,890) of isolates had both operons in full, while 55.8% (8,994) had a partial complement of *maf* genes in one or both operons (Fig. 3, Data File S2). The latter isolates were grouped and placed into the category ‘Other’ (full MAF2_5 operon and partial MAF6_7 operon, *n*=3,383; full MAF6_7 operon and partial MAF2_5 operon, *n*=1,300; partial for both operons, *n*=3,951; no genes of either operon, *n*=360). A small number of isolates were characterized as having either operon exclusively, 1.4% (235) for the MAF2_5 operon and 0.1% (11) for the MAF6_7 operon. Thus, flagellin glycosylation could possibly be achieved by a mixture of NCTC11168-like *maf* genes in the absence of full operons as found in *C. jejuni* strain NCTC11168.

### *maf3*, *maf4* and *maf5* are the core gene set of the *maf* gene family

**Fig. 4.**
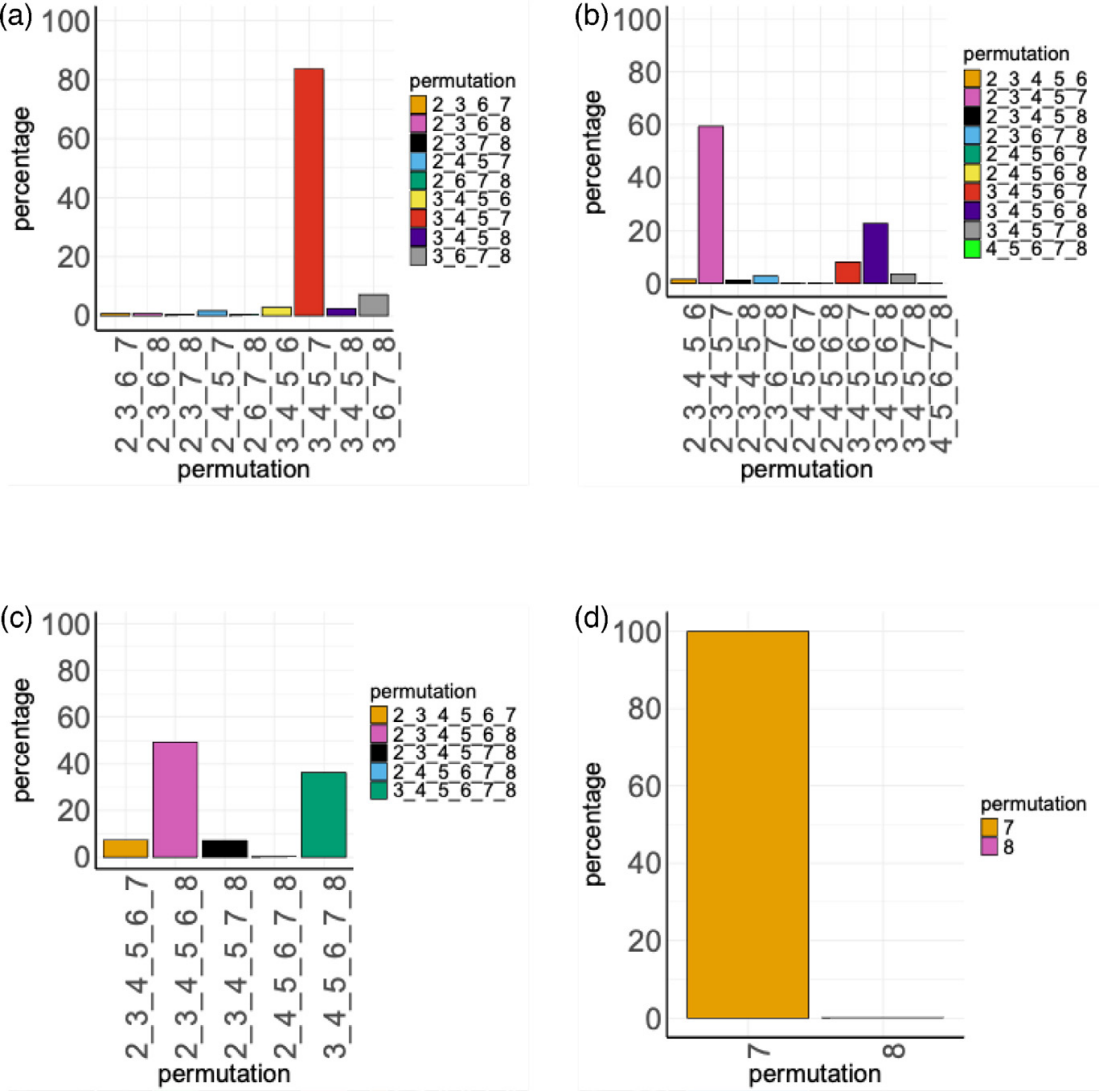
Gene permutations in isolates with a mixture of *maf* genes. For isolates where both of the full *maf* operons were absent, the presence of any permutation of the seven *maf* genes (*maf2–maf8*) was assessed (note that isolates lacking all genes are also excluded from this analysis). Isolates were sorted into subsets according to the number and type of *maf* genes present in the two *maf* operons. For example, a four-gene permutation may consist of *maf2*-*maf3*-*maf6*-*maf7* or *maf2*-*maf3*-*maf4*-*maf6*. Gene names are abbreviated to the relevant number for each gene (e.g. 2 is *maf2*). The four panels represent multiple permutations of a specific number of *maf* genes (*n*=number of isolates): (a) four genes (*n*=252), (**b**) five genes (*n*=2,009), (**c**) six genes (*n*=3,350) and (**d**) one gene *(n*=2,941)*. T*he number of isolates with each gene permutation was counted. Data represent counts as a percentage of the total number of isolates at each level of permutation. Note that while the assessment covered all possible permutations, only permutations with counts above zero are shown for each permutation group.

The presence of isolates with a mixture of *maf* genes suggested that the two *maf* operons could be reduced to smaller functional units in some isolates. Analysis of the 8,994 isolates with partial NCTC11168-like *maf* operons showed that the majority possessed one (33%, 2,941), five (23%, 2,009) or six (38%, 3,350) *maf* genes, while isolates with two to four *maf* genes were rarer (7%, 334, Fig. 4, Data File S2). The isolates with 0–3 *maf* genes may represent poor genome assemblies or could contain either novel genes or genes that have diverged significantly (i.e. below our 80% identity and/or 50% coverage cutoffs) from the strain NCTC11168 genes. For the larger units of the two partial operons, the simplest functional unit consisted of *maf3*, *maf4*, *maf5* and *maf7* and was present in 82% (211/252) of isolates that possessed four out of the six genes (Fig. 4a). Contrastingly, the five gene permutations displayed two independent streams (Fig. 4b). One stream (1,196 isolates) had *maf2* and *maf7* in addition to *maf3*, *maf4* and *maf5,* while the other stream (456 isolates) retained the latter 3 genes but substituted *maf2* and *maf7* with *maf6* and *maf8*. A similar substitution was also prevalent among six gene permutations where *maf*2 was replaced by one of *maf6, maf7* or *maf*8, while *maf3*, *maf4* and *maf5* were unchanged in each stream (Fig. 4c). These two streams accounted for 36% (1,213/3,350) and 49% (1,649/3,350), respectively, such that the *maf3*, *maf4* and *maf5* combination is a dominant gene set present in 85% of this group. Interestingly, although *maf7* was frequently substituted in the 4–6 gene permutations, this gene was found in 2,939 of the 2,941 isolates with only 1 *maf* (Fig. 4d).

### The *maf3* knockout is associated with a non-motile phenotype

**Fig. 5.**
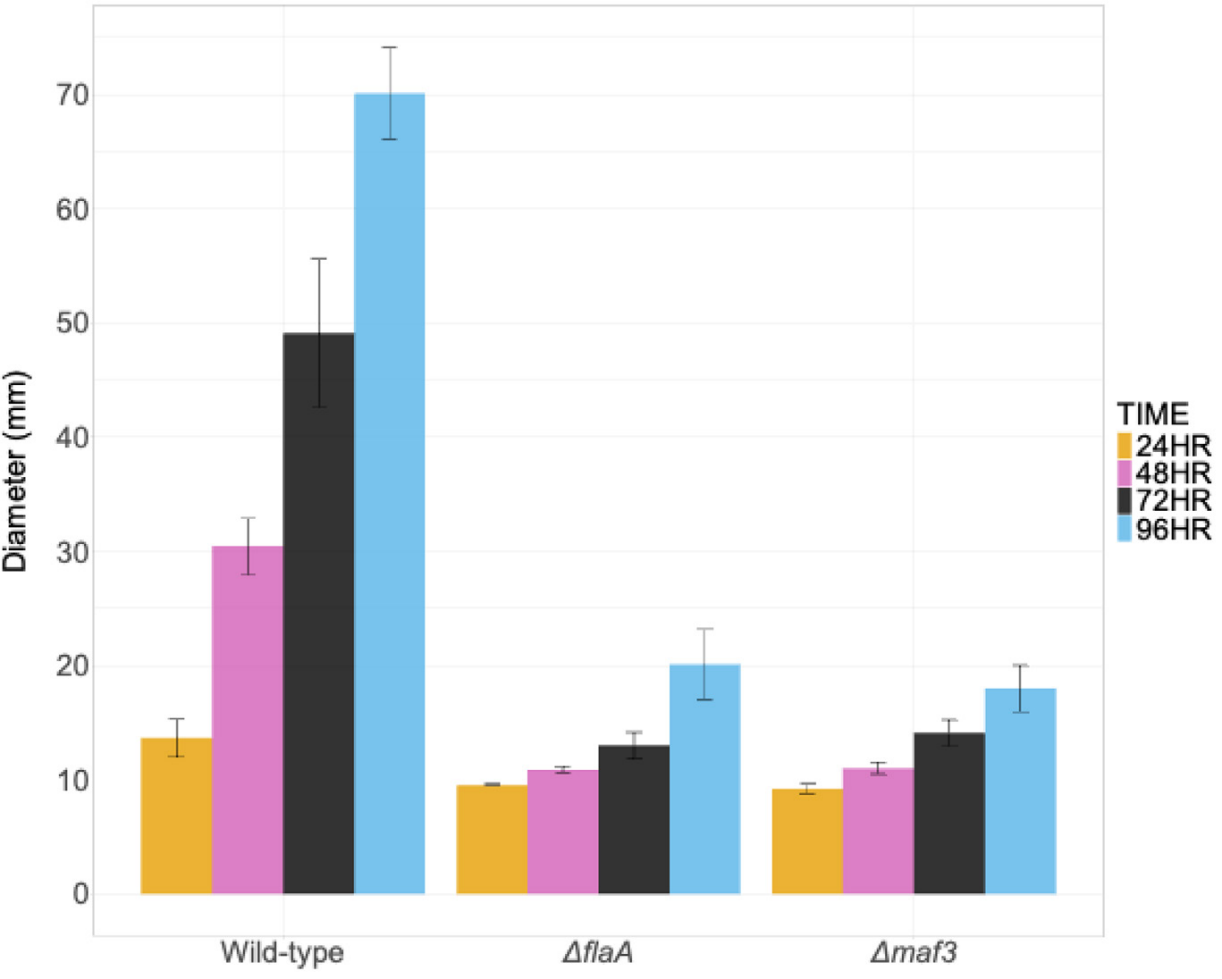
Motility in *C. jejuni* strain NCTC11168 wild-type isolate and isogenic mutants. *C. jejuni* strains and mutants were grown overnight on MHA plates and adjusted to 0.3 OD_600nm_. 0.4% (w/v) MHA plates were inoculated in the centre with 10 ul of bacteria and incubated at 42 °C under microaerobic conditions. The diameter (mm) of the halo was measured, and images were taken at each time point across 96 h. Data represent the mean distance for three biological replicates ±sem.

The bioinformatic analysis had shown that the core *maf* gene set comprised three genes, one of which is subject to PV (i.e. *maf4*) and another, *maf5*, results in non-motile organisms when deleted (see the ‘Introduction’ section). To investigate the functional effects of the third gene, *maf3*, a deletion mutant was constructed and tested for motility in the *C. jejuni* strain NCTC11168 background. The wild-type strain had a motile phenotype that reached a 70-mm halo diameter on day 4 post-inoculation (Figs 5 and S2). In contrast, a Δ*maf3* knockout mutant was non-motile, similar to the flagella knockout across 96 h of incubation (Figs 5 and S2). Complementation of the Δ*maf3* mutant by expression of *maf3* from the *metK* promoter restored motility to wild-type levels (Fig. S2). Thus, deletion of one of the non-phase variable genes of the core gene set can result in a non-motile phenotype. However, motile variants were readily obtained at late time points in motility experiments (Fig. S3), indicating that the *maf3* mutant phenotype is context-dependent.

### Electron microscopy reveals the aflagellate nature of the *Δmaf3* mutant

**Fig. 6.**
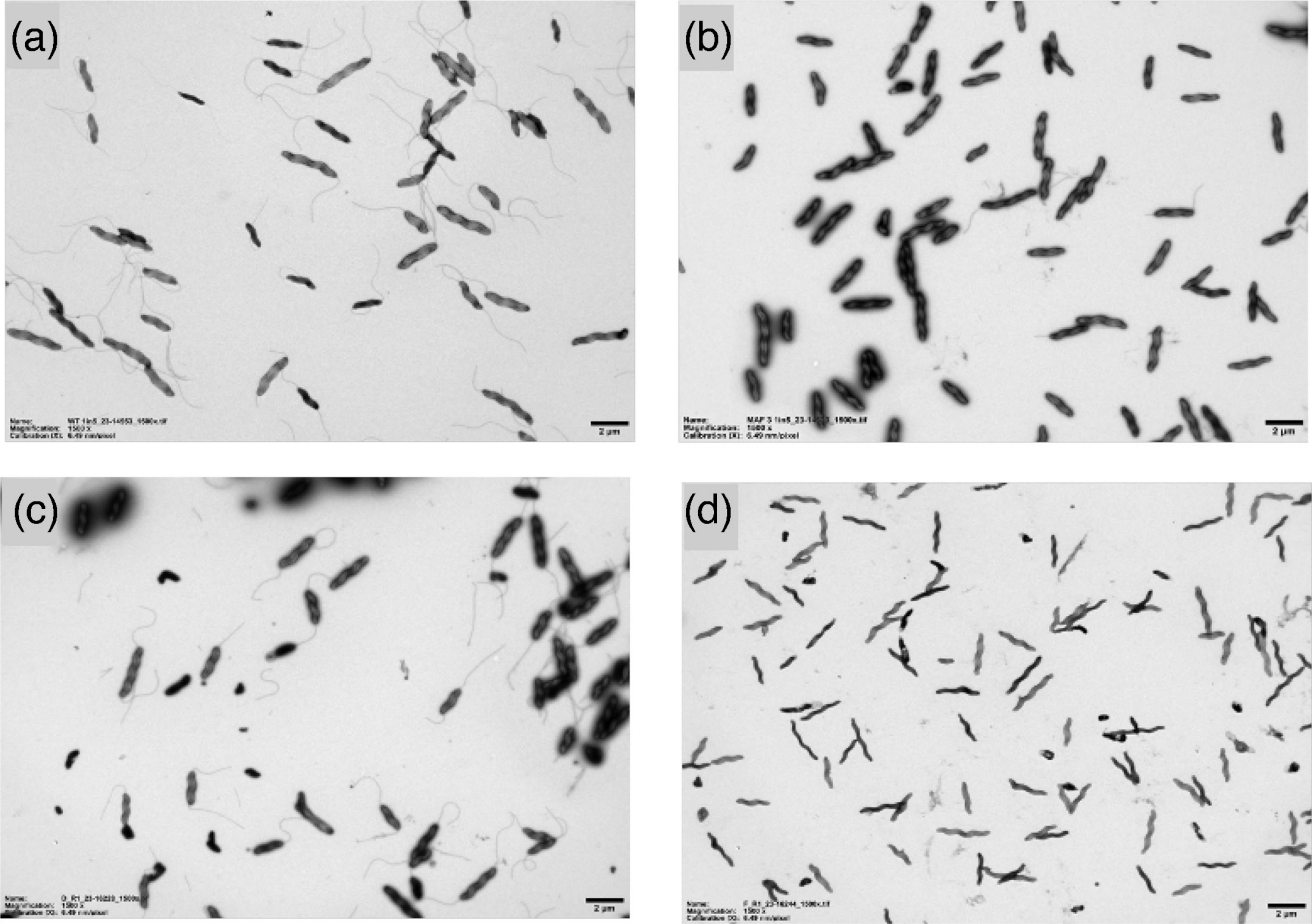
Filament formation is affected by the *maf3* glycosyltransferase in *C. jejuni* strain NCTC11168. After 16 h growth, bacterial cells were pelleted, washed with 1× PBS and resuspended in 1× PBS containing 2.5% glutaraldehyde to an OD_600_ of 1.0. Cells were negatively stained and then immediately subjected to analysis on a JEOL JEM-1400 TEM electron microscope with an accelerating voltage of 120 kV. Digital images were collected with an EMSIS Xarosa digital camera with Radius software. (a) Wild-type, (b) *Δmaf3*, (c) *Δmaf3*::metK-*maf3* and (d) *ΔflaAB*.

Flagella-modifying genes can affect filament formation through post-translational modification of flagellin protein. Since filament formation is a result of monomer polymerization, mutants in these genes may lead to flagella instability, truncation or complete absence of the filament. The flagella filament was examined by electron microscopy to assess the effects of gene deletion on filament formation (Fig. 6). The wild-type strain was characterized by the canonical spiral shape of *C. jejuni* and polar flagella (Fig. 6a). The *Δmaf3* mutant had a wild-type cellular morphology but no polar flagella, as also observed for the *flaA/B* double knockout mutant (*ΔflaAB*), Fig. 6b, d, respectively. Polar flagella were restored upon complementation of the Δ*maf3* mutant (Fig. 6c). Notably, some of the Δ*maf3* mutant cells had polar flagella, which likely reflects motile revertants as detected in motility experiments.

### Phase variation underpins the motile phenotype observed in Δ*maf3* mutant variants

**Fig. 7.**
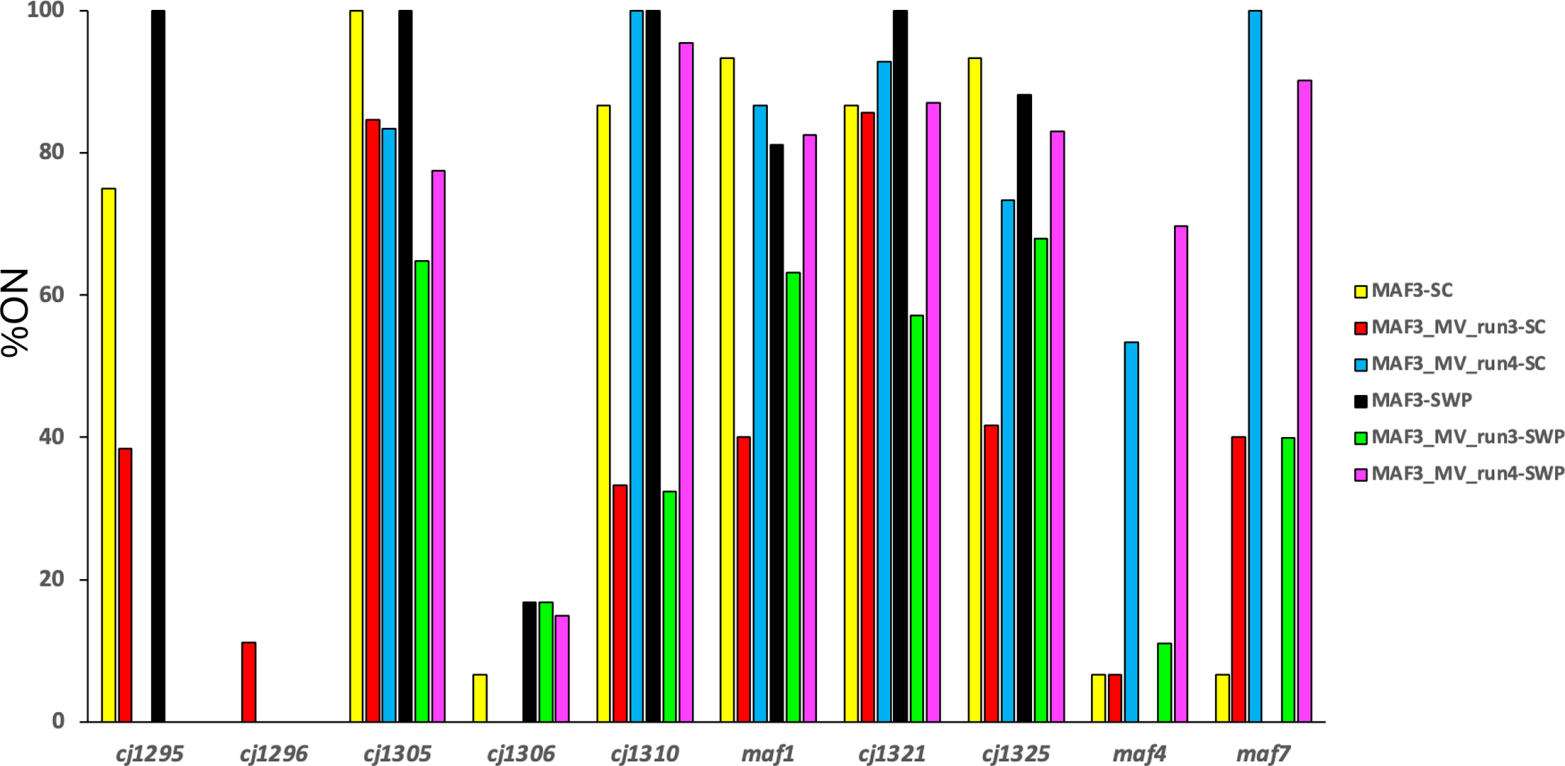
Phase variation states for *maf* genes in Δ*maf3* mutants and motile revertants. GeneScan analysis was performed on the *Δmaf3* mutant and the *Δmaf3* motile variant (MV). Motile variants were obtained by sampling from outgrowths at the edge of motility plates. Strains were re-plated from glycerol stocks and analyses were performed either on sweeps (SWP) from low dilution plates or single colonies (SC) (*n*=12–15) sampled from high dilution plates. Percentage ON/OFF states were determined from GeneScan analyses of samples relative to a genomic DNA preparation of known repeat number and expression state for each gene. For sweep data, the %ON state was calculated by dividing the peak areas of the ON state peak by the total area of all peaks. For colony data, the %ON state was calculated as the percentage of colonies with an ON state divided by the total number of colonies.

*C. jejuni* strain NCTC11168 has 28 phase variable loci with 10 located in the glycosylation locus [11,20] (Fig. 1). PV of some of these genes is associated with changes in flagella glycosylation states and phenotypes (see the ‘Introduction’ section). To assess whether PV was responsible for the reversion to motility of Δ*maf3* variants, GeneScan analysis of the ten glycosylation locus PV genes was performed on the Δ*maf3* mutant and Δ*maf3* motile variants recovered from the edge of the halo at 96 h post-inoculation for two of the four motility assays (Fig. 7). For this analysis, GeneScan was performed on 12–15 single colonies and sweep samples (Fig. S4). As an additional confirmation, GeneScan analyses were also performed for five of these PV genes on the inoculum, non-motile core variants (i.e. bacteria isolated from around the inoculation area of the motility plate) and motile variants sampled from the edge of the halo (Fig. S4).

The single colony analysis showed that run 4, but not run 3, motile variants had switched to high ON states for *maf4* and *maf7* as compared to the parental Δ*maf3* mutant (Fig. 7, Data File S3). No consistent changes were observed in any other genes, with some genes either consistently OFF (e.g. *cj1296*) or ON (e.g. *cj1305*). The sweep PV analyses showed that *Δmaf3* mutant inoculums and core variants had similar profiles apart from a ~60% reduction in the *cj1325* ON state (Fig. S5, Data File S4). Again, motile variants from run 3 had similar profiles to inoculum samples and core variants. Contrastingly, run 4 motile variants had expanded the ON states of both *maf4* (63%) and *maf7* (100%) as compared to the inoculum and core variants. These differences could be incidental but alternatively may explain preliminary observations of higher motility of Δ*maf3* run 4 motile variants as compared to run 3 variants.

### *Δmaf3* motile variants are associated with a *maf5* SNP

As reversion to or loss of motility has been associated with point mutations in *C. jejuni* flagella genes, whole-genome sequencing (WGS) was performed to assess the possible relevance of other mutations to observed phenotypes of mutants, complementation strains and the run 4 motile variants. Using the *C. jejuni* strain NCTC11168 (NC_002163) genome sequence as a reference, our chicken-adapted wild-type strain was found to have several genetic differences as compared to this published genome (Data File S5). These variants were, however, shared across all isogenic mutants and variants and so were excluded from further analysis. Alterations were noted in multiple PV tracts, with three being specific to the motility variant (*cj0031*, *cj1295* and *cj1426*). However, these alterations may have occurred as part of the WGS analysis. Notably, the run 4 motile variant had a unique mutation in the coding sequence for *maf5* (*cj1337*; p*seE*; 1266528G-to-A, Data File S5). This polymorphism in *maf5* was non-synonymous, producing a glutamate (GAA) to lysine (AAA) alteration that may affect the function of this important flagellin glycotransferase and possibly contribute to the reversion of the non-motile *maf3* phenotype.

## Discussion

The genome sequence of *C. jejuni* strain NCTC11168 has a large flagella locus that includes genes encoding two flagellins, biosynthetic pathways for two nonulosonic acids and seven motility accessory factors (*maf*) [11]. Several genes in this locus were also found to have homopolymeric tracts that can generate glycan heterogeneity on the flagellin proteins through PV [10]. Biosynthetic and functional phenotypes in a range of *C. jejuni* strains have identified functions for some of the *maf* genes; however, distributions for these genes across the species have not been investigated in detail and could improve our understanding of the roles of these genes in motility and other functions of *Campylobacter* flagella.

**Fig. 8.**
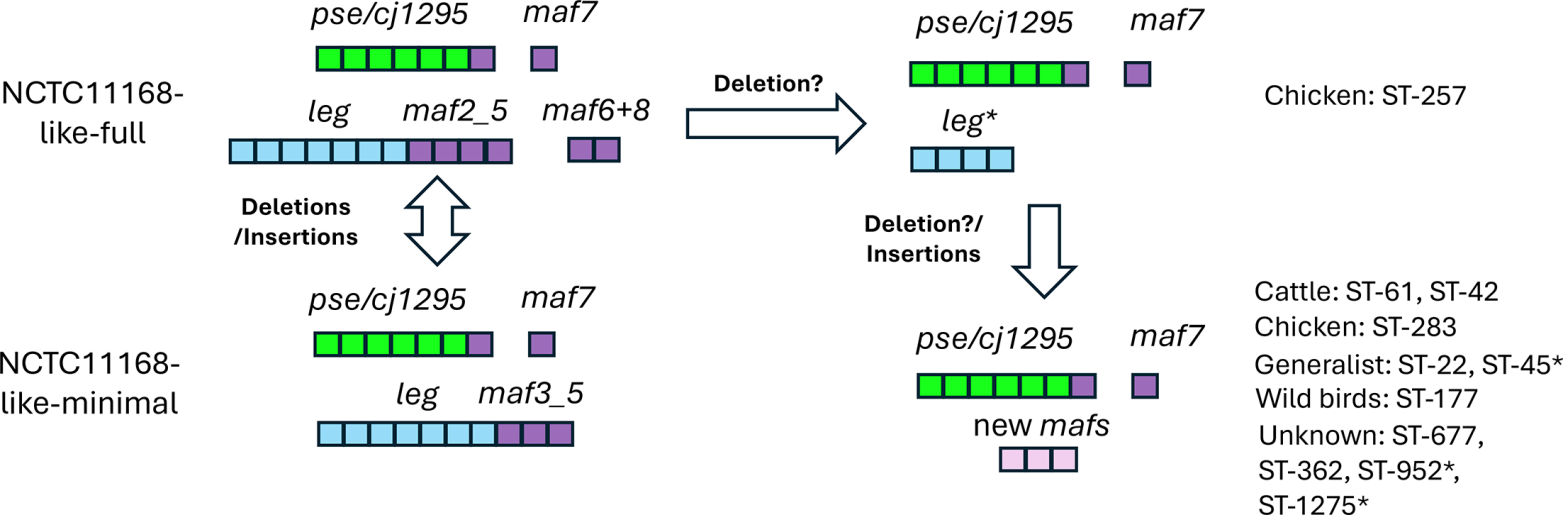
Hypothetical model of the evolution of the *C. jejuni* flagella locus. This figure summarizes the distribution patterns of the NCTC11168-like *maf* genes among 16,130 strains and 37 clonal complexes of *C. jejuni*. The NCTC11168-like-full pattern refers to the full complement of *pse*, *leg* and seven *maf* genes of *C. jejuni* strain NCTC11168 (see Fig. 1). The full complement of NCTC11168-like *maf* genes was found in 42.7% (*n*=6,890) of strains. The NCTC11168-like-minimal pattern refers to strains where the full *pse* and *leg* gene sets are present but where homologues of the NCTC11168 *maf2*, *maf6* and *maf8* genes are absent. Note that genes with homology values below our cutoffs may be present and that multiple patterns of presence/absence exist between the full and minimal gene set. In contrast, eleven clonal complexes exhibit full or partial deletions of the *leg* genes and the *maf2–5* operon in either all strains or a substantial number of strains (with the latter marked by an asterisk). These strains may contain novel *maf* genes that have limited homology to NCTC11168-like *maf* genes (see text and Fig. 9). We postulate that the loss of the *leg* genes may have occurred as one or more deletion events in a founder strain.

Bioinformatic analysis of the glycosylation locus across 16,130 *C*. *jejuni* isolates showed a correlation between homologues of the NCTC11168 flagellin-modifying genes and the pseudaminic acid and legionaminic acid synthesis gene clusters (Fig. 8). When legionaminic biosynthesis genes were present at very low prevalences (0–0.04%; 22, 42, 61, 177, 283, 257 and 677) or were of reduced presence (0.4–0.8%; 45, 952 and 1275) in a clonal complex, *maf1–6* genes also had a very low or reduced presence, respectively (Fig. 2). A similar correlation between the presence of legionaminic acid biosynthesis and one of these *maf* genes, *maf4*, was recently observed in a bioinformatic analysis by Meng *et al*. across 258 *C*. *jejuni* genomes. These authors also performed metabolic oligosaccharide engineering work, which showed that *maf4* was a putative legionaminic acid transferase in *C. jejuni* strains 108 and RM1221 [15]. Our work expands this genomic analysis to include multiple *C. jejuni* glycosylation locus genes and clonal complexes, suggesting that *maf* and *leg* gene associations are a widely distributed phenomenon among *C. jejuni* strains.

**Fig. 9.**
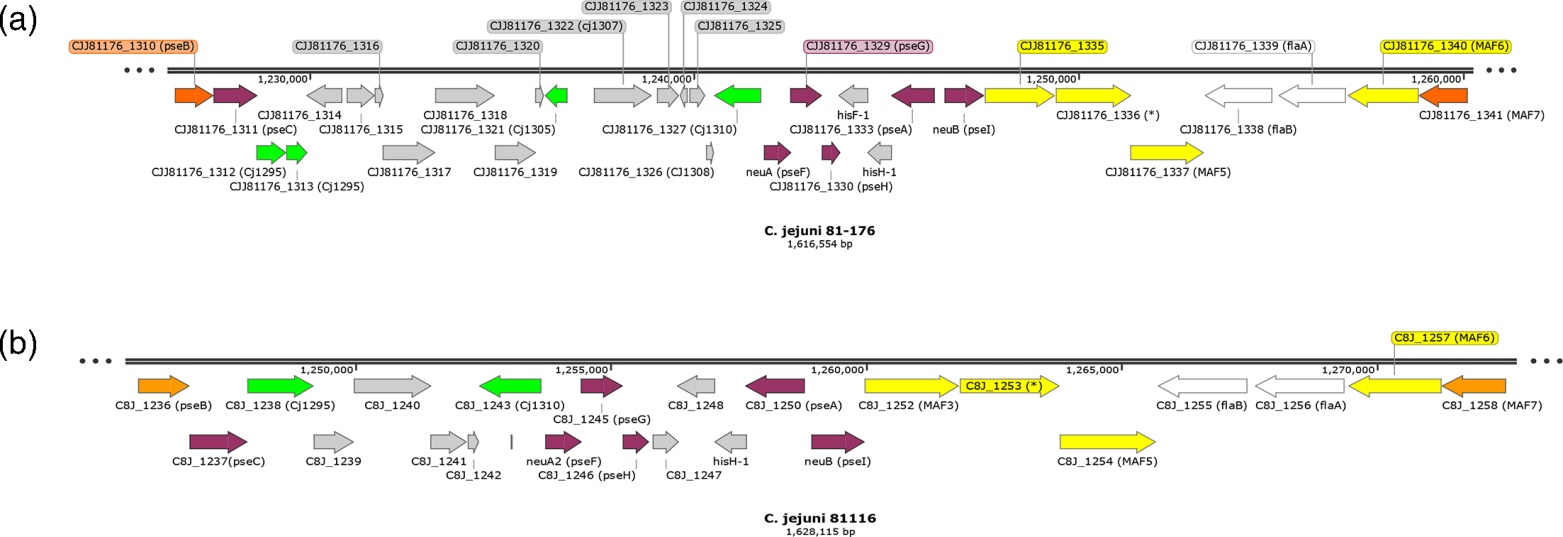
Cluster of flagella structural, biosynthetic and modification genes in the genome sequences of *C. jejuni* strains that lack legionaminic acid biosynthesis genes. This diagram depicts the flagella glycosylation locus found in *C. jejuni* strains 81-176 (a GenBank: CP000538.1; ST-42) and 81116 (b GenBank: CP000814.1; ST-283). Gene sequences from each strain were used to perform tblastn against the *C. jejuni* strain NCTC11168 genome. Genes are shown as arrowed rectangles and coloured according to the *C. jejuni* strain NCTC11168 homologues, whose names are in parentheses and, except *maf5*, were identified based on a tblastn identity of >70%. The *maf5* homologues had tblastn scores of 56% and hence are weak homologues of the NCTCT11168 *maf5* gene. The colour scheme follows that of Fig. 1*.* Images were rendered in SnapGene version 8.2.1.

Of the seven clonal complexes with very low prevalences of *leg* biosynthesis genes, the ST-61 and ST-42 complexes have been designated as host specialists that are strongly associated with cattle [22]. Two other groups, Zang *et al*. and An *et al*., have also reported a low prevalence of *maf4* gene sequences and a high prevalence of pseudaminic acid biosynthesis genes, respectively, across genomes from cattle isolates [23,24]. An *et al*. postulated that the significantly high levels of pseudaminic acid genes in cattle isolates might be due to the dense mucosal environment acting as a selective pressure for robust flagella, thus driving host-specific adaptations [24]. Contrastingly, Deblais *et al*. found *legG* in 38/56 genomes isolated from dairy cattle dung; however, this high prevalence might have been driven by high numbers of generalist (e.g. ST-21 complex, *n*=20) strains rather than host specialists (e.g. ST-61 complex, *n*=7) since isolates covered multiple clonal complexes [25]. Overall, these results suggest that the cow gastrointestinal tract may favour predominantly pseudaminic acid-modified flagella over legionaminic acid modifications.

The importance of legionaminic acid modifications for chicken colonization has been shown by two groups. Howard *et al.* [26] showed that deletion of *cj1321–cj1326* and *cj1324 (ptmG*) alone reduced chicken colonization with the latter mutant lacking legionaminic acid flagella modifications [26]. Zebian *et al*. [27] found that a *cj1319* (*legB*) had higher levels of legionaminic acid flagella modifications and enhanced chicken colonization [27]. Our observation that 82% (9/11, Fig. 2) of chicken specialist clonal complexes have a high prevalence of legionaminic acid biosynthesis and *maf* glycan-modifying genes suggests that these associations may be a general feature of these host specialist lineages. This association is further underpinned by the observation that 71% (5/7, Fig. 2) of generalist clonal complexes also have a high prevalence of these two gene sets. However, the presence of legionaminic modifications in these latter isolates could have additional benefits for environments outside the host. For example, some *Bacillus subtilis* spores are covered with a crust layer that requires legionaminic acid for proper assembly, with the absence of the crust layer altering surface properties and spore adhesiveness [28,29]. Additionally, Pinel *et al.* detected pseudaminic acid and legionaminic acid in environmental biofilms, with productions of these sialic acids being maintained even under nutrient starvation conditions [30]. In *Campylobacter*, the lack of legionaminic acid modification on flagella leads to reduced interaction with *Acanthamoeba castellanii* and significant reductions in intracellular bacteria [31]. *A. castellanii* has been hypothesized to be a transient host for *C. jejuni* and other bacteria offering refuge to non-spore-forming bacteria between hosts [32,33]. These findings highlight potential properties that legionaminic acid may confer on bacterial pathogens during persistence outside of their major hosts and may be a factor in the high prevalence of *leg* genes among generalist clonal complexes of *C. jejuni*.

A key finding of our genomic analyses was that *maf* genes were often present as subsets of genes and operons (Figs 2–4). Central to these permutations was a dominant profile (>80% of isolates) where *maf3*, *maf4* and *maf5* were always present, even in isolates with a minimal NCTC11168-like *maf* gene set (Fig. 8). As we discuss below, these genes may represent a core NCTC11168-like *maf* gene set. This core gene set was then associated with an auxiliary set of other NCTC11168-like *maf* genes: *maf2* only, *maf7* only or *maf6* and *maf8* only. These correlations suggest that the presence of legionaminic acid biosynthesis genes drives the presence of the NCTC11168-like *maf* flagellin-modifying genes, with these genes being required for the addition or modification of legionaminic acid moieties, as is already known for *maf4* [15]. This association may also be driven in part by the close genomic association between *maf2–5* and *leg* biosynthetic genes, with all of these genes being acquired as part of a large gene cluster (Fig. 1).

*C. jejuni* strains 81-176 and 81116 are representative of two of the clonal complexes, ST-42 and ST-283, respectively, where the NCTC-like *maf* and *leg* genes are absent (Fig. 2). Examination of the organization of the flagella locus (Fig. 9) and our nucleotide blast data outputs (Data File S6) for these strains indicated that homologues of the NCTC11168 *maf3–7* genes are present but with low nucleotide identity scores for *maf3–6* (68–78%). Conversely, *leg* gene homologues are absent in these strains with no significant homology matches. This may represent a deletion event, as only 7 of the 38 clonal complexes consistently lack the *leg* genes. To examine functionality, amino acid sequence analyses were performed by tblastn, and identity scores of 93–99% were obtained for the Pse proteins (Data File S6). These genes are likely to have highly conserved functions. Conversely, amino acid identity scores for the Maf2, Maf3, Maf5 and Maf6 amino acid sequences were between 56 and 71%, indicating that these genes are likely to be functionally divergent. This suggests that there is a set of NCTC11168-like *maf* genes and a set of divergent *maf* genes, likely with novel functions. The concomitant absence of the NCTC11168-like MAF2_5 operon and *leg* genes might suggest a functional link. So far, research has shown that *maf5* is not involved in the synthesis/modifications of glycans but in the attachment of glycans to flagellins, specifically, pseudaminic acid and its derivatives, with *maf5* mutants being non-motile [8]. However, whether or not *maf5* can also transfer legionaminic acid residues to flagellins remains to be determined. The recent discovery of *maf4* as a putative legionaminic acid transferase [15] and our analysis showing a core maf gene family (*maf3*, *maf4* and *maf5*) that correlates with the presence of legionaminic acid biosynthesis genes suggests that the activity of these three *maf* glycotransferases in relation to each other and attachment of legionaminic residues to flagellins is yet to be fully elucidated.

Our large-scale genomic analysis of *Campylobacter* strains should be considered in the context of potential biases arising from particular sequencing methodologies and deficiencies in assembly of complex regions [34]. Biases in coverage of specific regions in *Campylobacter* and other low GC content genomes can occur when using certain types of library preparation kits [35]. Read length and depth can also affect coverage and assembly, particularly for multicopy genes. Methodological information and sequence metrics are not available and/or readily extractable from PubMLST databases. However, we were able to access other proxies for genome sequence quality, which showed that all genomes had 200 or fewer contigs and genome sizes of between 1.6 and 2.1 MBp (Fig. S6 and Data File S1). These values are within the bounds utilized for similar *Campylobacter* genomic analyses [36,37]. To further ascertain whether genome quality influenced our results, we examined the flagella gene distributions utilizing contig number as a measure of the accuracy of genome assembly. The patterns of presence/absence of the *maf1–6* and *leg* genes were consistent for genome sequences consisting of 1–10, 11–50 and 51–200 contigs, indicating that sequence quality and genome assembly were not biassing observations on gene distributions (Fig. S7). We also note that the proportions for each ST complex are averaged from data from multiple sequencing projects, thereby reducing the potential for ST-specific biases arising from methodological factors.

The phenotype of *maf3*, one of the core *maf* genes, had not previously been explored. Biological assays showed that a Δ*maf3* knockout had a non-motile phenotype and no flagella filament formation (Figs 5, S2 and S6). This mutant was non-motile despite a high percentage of ON states for many of the PV genes in the flagella glycosylation locus including *maf1* (Fig. 7), which was previously shown to rescue motility in a *maf5* knockout mutant [10]. The absence of a filament in the *Δmaf3* mutant is indicative of a failure in flagellin polymerization (Fig. 6). As polymerization is thought to be dependent on flagellin glycosylation by pseudaminic acid in *C. jejuni* strains, this might suggest that *maf3* is involved in the transfer of pseudaminic acid to the flagellin. However, similar to *maf5* mutants, motile revertants were easily obtained, possibly suggesting some crosstalk between pseudaminic acid and legionaminic acid biosynthetic pathways for regulating or promoting filament formation.

Examination of two Δ*maf3* motility revertants provided some indications of possible interactions between the *maf* genes. The run 4 motility revertant was found to be flagellated during motility assays (Fig. S3) and exhibited switching of *maf4* and *maf7* into high ON states (Figs 7 and S5). Surprisingly, *maf1*, responsible for conferring motility on non-motile Δ*maf5* mutants [10], was already ON in the inoculum. As *maf1* and *maf4* have identical sequences in *C. jejuni* strain NCTC11168, this may suggest that there is a gene dosage effect or that expression of these genes is regulated independently of PV. This revertant also had a missense mutation in *maf5*. It is possible that this non-synonymous mutation altered the function of *maf5* either reducing or altering the function of this gene to add a different type of sugar. In *C. coli* strain VC167, only a double knockout of *pseB* and *ptmD* resulted in a non-motile phenotype, which showed that either pseudaminic or legionaminic acid modifications are capable of supporting flagella assembly [7]. The lack of motility of *maf3* and *maf5* mutants and reversion of motility by compensatory changes may indicate that *pse* or *leg* modifications have inhibitory or compensatory effects on flagellin elaboration in *C. jejuni*. Biochemical analyses of the *maf3* and *maf5* genes are required to elucidate how these genes are involved in the regulation of motility in *C. jejuni*.

Motility is essential for host colonization by *C. jejuni* [5], and hence, the potential for legionaminic acid to compensate for pseudaminic acid shutdown would be evolutionarily advantageous for maintaining flagella filament formation in this species and other *Campylobacter* species [7]. *C. jejuni* strain NCTC11168 is a strain that has both sets of biosynthetic genes and could employ this strategy in maintaining flagellar structural integrity. Our genomic analysis has shown that the *maf* genes of the NCTC11168 strain are widely distributed among *C. jejuni* strains and lineages and that these genes are organized into core and auxiliary gene sets. Our analysis of a *maf3* mutant adds to previous work showing that these core genes can generate reversible, non-motile phenotypes. Further exploration of the biochemical functions of the core *maf* genes and genetic analyses of the auxiliary genes are required to further unpack the black box of *Campylobacter* flagella modification.

## Supplementary material

10.1099/mic.0.001698 Supplementary Data Sheet 1.

10.1099/mic.0.001698 Uncited Supplementary Data Sheet 2.

10.1099/mic.0.001698 Uncited Supplementary Data Sheet 3.

10.1099/mic.0.001698 Uncited Supplementary Data Sheet 4.

10.1099/mic.0.001698 Uncited Supplementary Data Sheet 5.

10.1099/mic.0.001698 Uncited Supplementary Data Sheet 6.

10.1099/mic.0.001698 Uncited Supplementary Material 1.

## Abbreviations

PV: phase variation
VAIN: variable atmosphere incubator
WGS: whole-genome sequencing
