# Supplementary material for: Identification of a core set of Campylobacter jejuni flagella modification genes and a reversible non-motile maf3 phenotype
Source: Microbiology (Reading). 2026 Apr 28;172(4):001698. doi: 10.1099/mic.0.001698 (PMC13130089; doi:10.1099/mic.0.001698)
Supplement: Uncited Supplementary Material 1. [file mic-172-01698-s007.pdf]

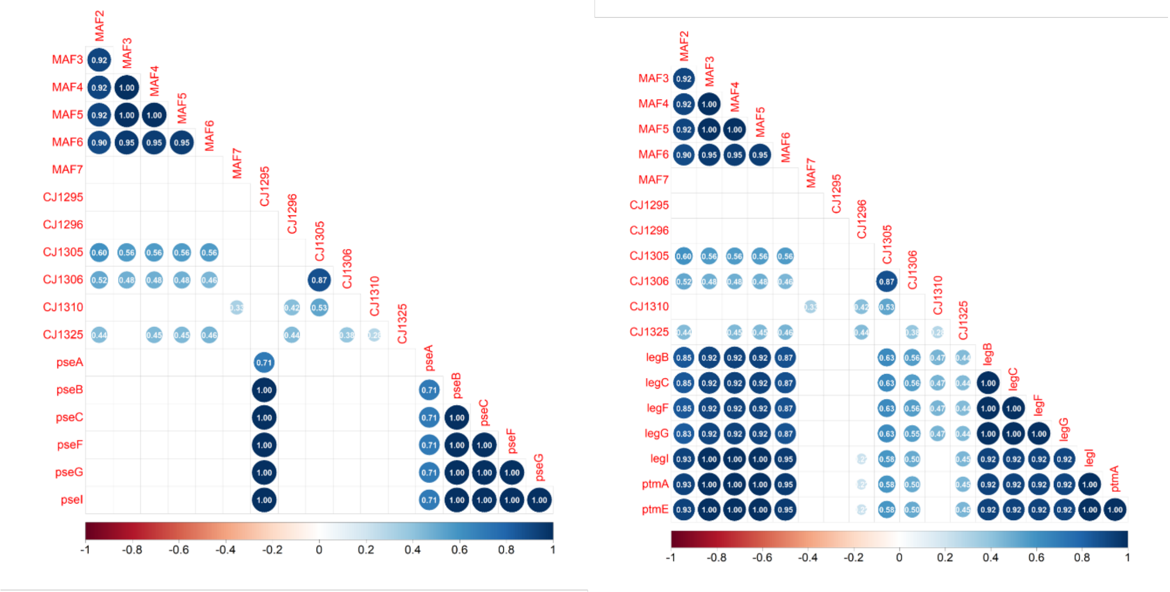

Figure S1. The presence of glycotransferase genes correlates with the presence of glycan synthesis genes. Spearman correlation analysis of presence/absence data for pseudaminic acid (left) and legionaminic acid (right) heatmaps respectively. Correlations ranged from negative (-1, red) to positive (1, blue). Significant coefficients are denoted by circles with size and colour intensity increasing as a function of the divergence of the coefficient from zero. Blank squares represent nonsignificant correlations, $p<0.05$.

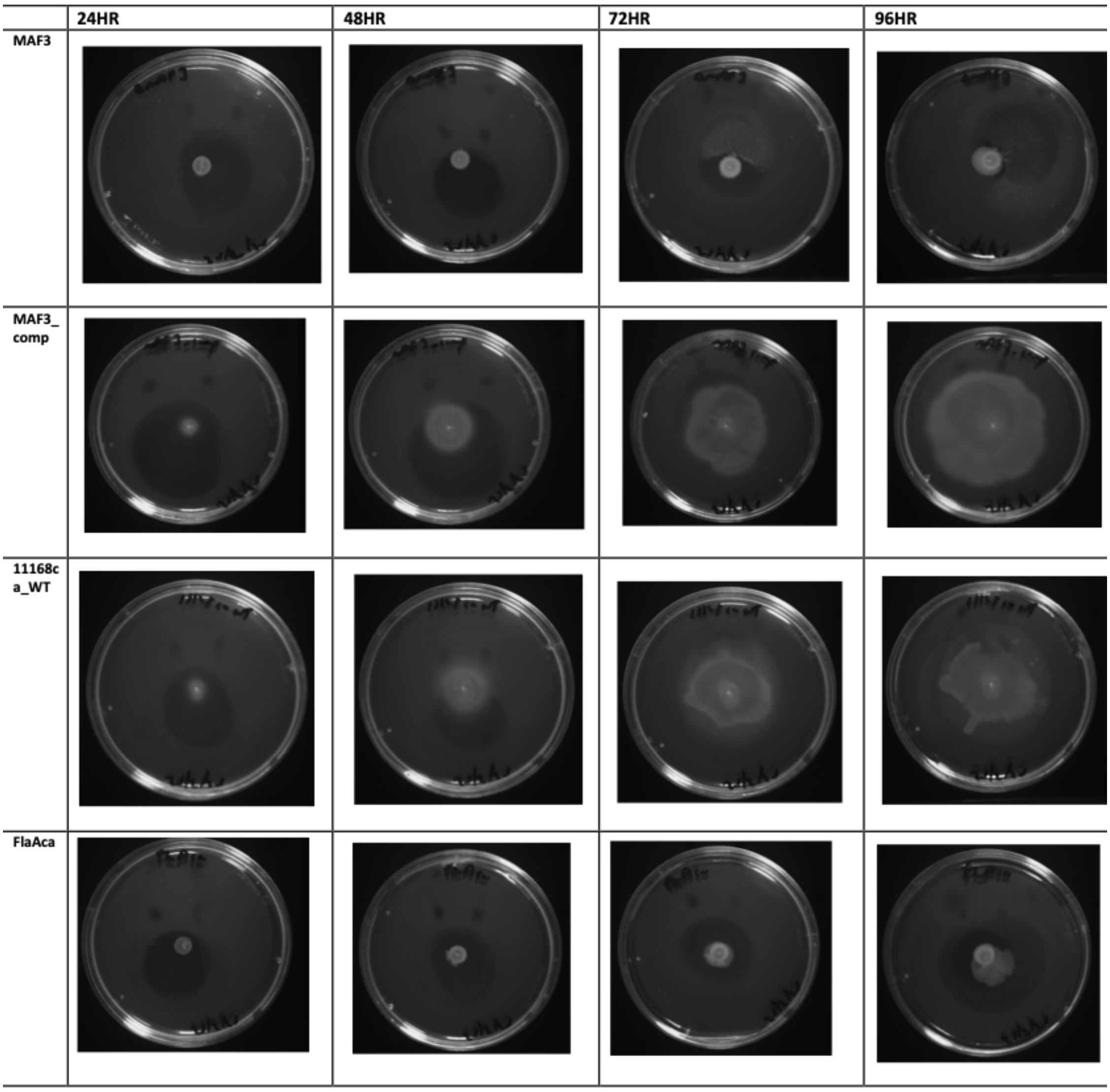

Figure S2. Swarming morphology in *C. jejuni* and isogenic mutants. This figure compares the swarming motility of the chicken-adapted (ca) variant of *C. jejuni* strain NCTC1168 (third row), two isogenic mutants with deletions in the *maf3* (top row) or *flaA* (bottom row) genes and a complementation mutant for *maf3* (second row). The *C. jejuni* NCTC11168 Δmaf3-kan^{R} mutant was subject to complementation by insertion of the *maf3* gene downstream of the *metK* promoter in *cj0046* complementation locus (which contains a chloramphenicol cassette for selection of transformants). *C. jejuni* strains/mutants were grown overnight in MHB broth followed by adjustment of the culture to 0.3 OD_{600nm}. Bacterial suspensions (10uL) were inoculated into the centre of 0.4% (w/v) MHA plates and incubated at 42°C under microaerobic conditions. The diameter (mm) of the halo was measured at 0, 24, 48 and 96 hrs. Images for the four time points (left to right, respectively) were also taken at each time point.

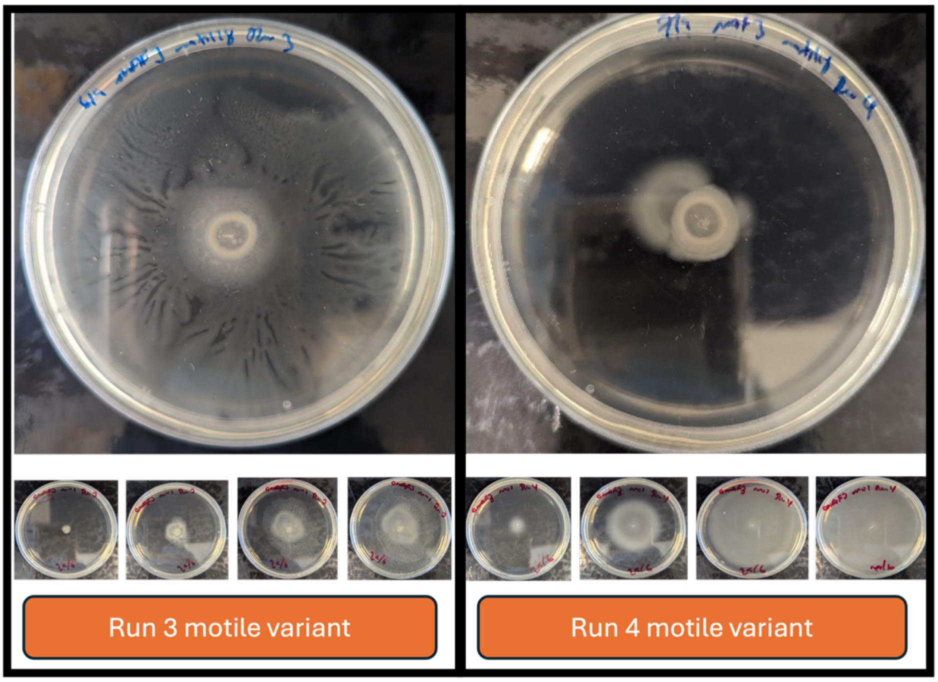

Figure S3. Swarming morphology of maf3 motility variants sampled from during motility assay runs 3 and 4. Swarming assays were performed as described in Figure S2. The Δ*maf3* motile variants were obtained from swarming out growths as can be observed in the top two pictures for run 3 and run 4. One motile variant from each of runs 3 and 4 were subject to re-analysis of their motility. Top row, pictures of Δ*maf3* motility run 3 and run 4 at 96 hrs post inoculation; these plates were the sources for the motility variants. Motile variants were stocked at -80°C and then retested in additional motility runs. Bottom row, pictures of the motility assay for each motility variant, Run 3 and Run 4 motility variant, after 0, 24, 48 and 96 hrs of incubation (left to right panels).

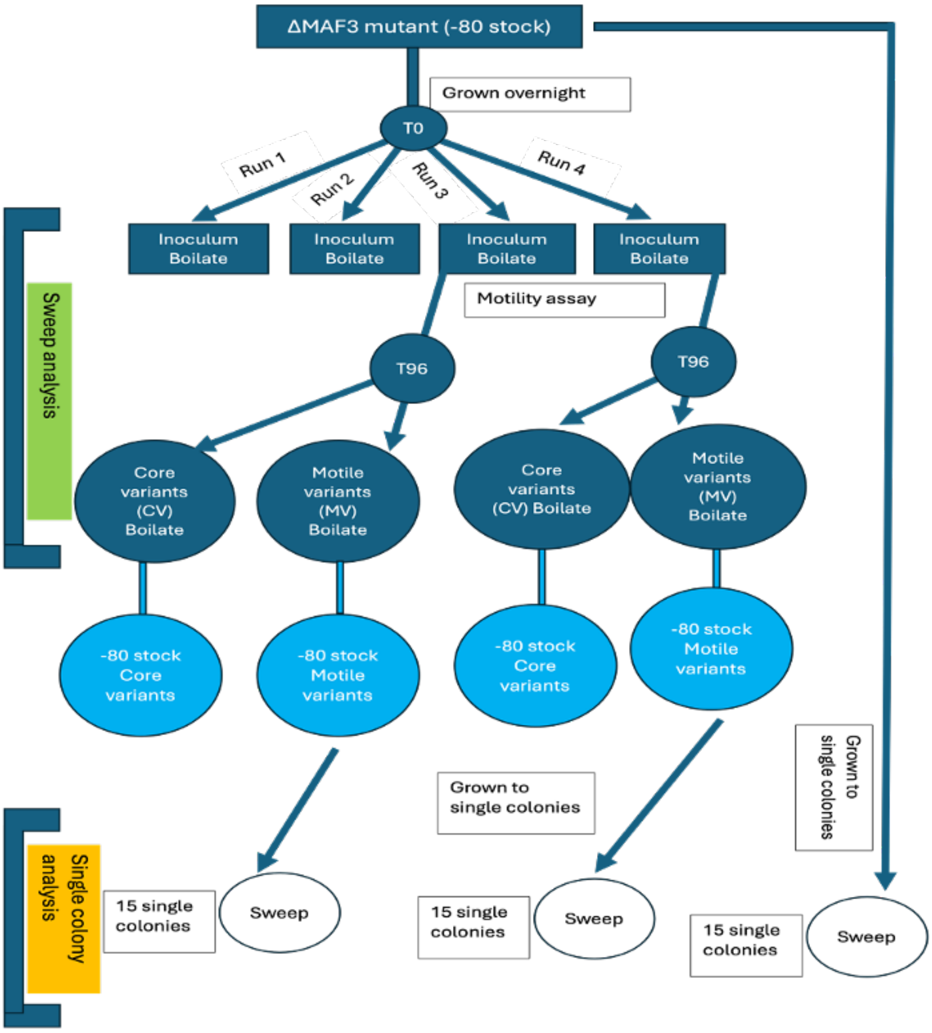

Figure S4. Illustration of sample processing for analysis of the phase-variable genes by GeneScan. This diagram shows a flow chart for the generation of samples for phase-variable gene analyses for the Δmaf3 mutant as an example. Four runs were performed. Inoculum populations were generated for all four runs but the analysis of core and motile variants was only performed for runs 3 and 4. Bacteria were grown overnight and adjusted to an OD600nm of 0.3 in MHB. An aliquot of 10uL of this suspension was used for the motility assay while another 50 uL aliquot was used to prepare a DNA boilate of the inoculum (T0 inoculum sample) for PCR and GeneScan analysis. After 96hrs, Δmaf3 motile variants were isolated from the centre (core variants; CV) and edges (motile variants; MV) of the halo on motility agar plates. These isolates were inoculated onto blood agar plates, incubated overnight and then utilized for preparation of boilates and -80°C stocks. For both the inoculum and T96hr boilates, sweeps of multiple bacterial cells were collected for use in a sweep analysis. Three isolates (Δmaf3 mutant, Δmaf3_MV_Run3 and Δmaf3_MV_Run4) were regrown from frozen stocks following streaking to yield single colonies. A total of 15 colonies and a sweep were collected for each isolate. Both sweep and single colonies were used for GeneScan analyses of phase-variable gene expression states.

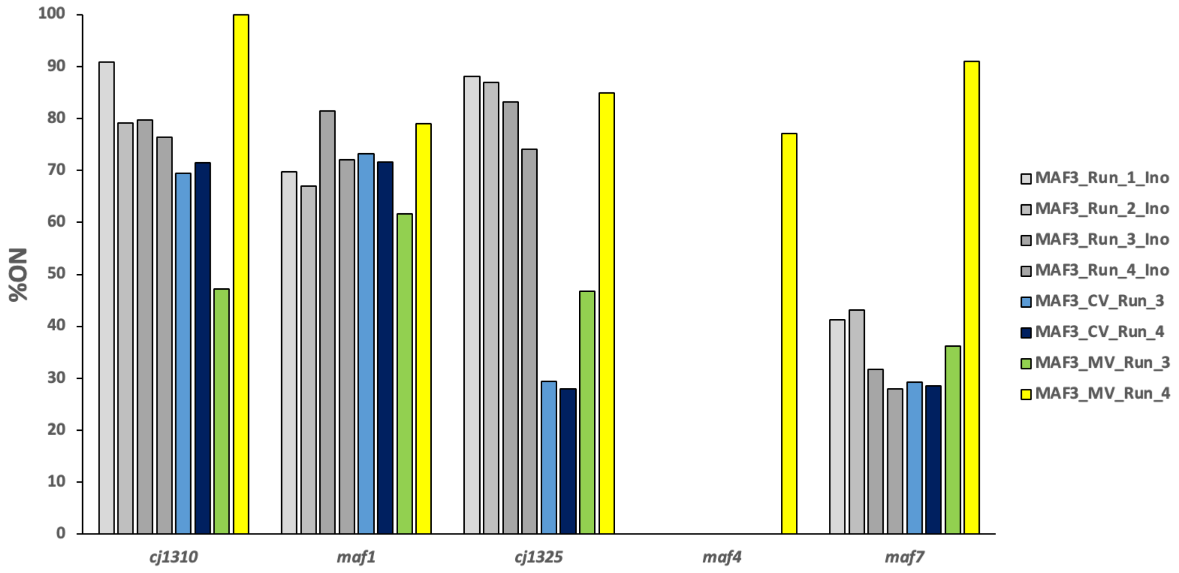

Figure S5. Phase variation states for *maf* genes for Δ*maf3* mutants and motile revertants. GeneScan analysis was performed on *C. jejuni* strain NCTC11168:Δ*maf3* mutant inoculum boilates (Ino) and on biolates of sweeps of samples obtained from either the centre of motility plates (CV) or the edge of the motile area. The bacteria obtained from runs 3 and 4 were subsequently shown to have re-gained motility and hence were termed motile variants (MV). Sample handling is detailed in Figure S4. GeneScan data was obtained for five of the genes from the NCTC11168 *fla* locus. The %ON state was calculated by dividing the peak areas of ON state peak by the total area of all peaks for each gene. The *maf* genes are:- *cj1318* (*maf1*), *cj1335* (*maf4*) and *cj1342* (*maf7*).

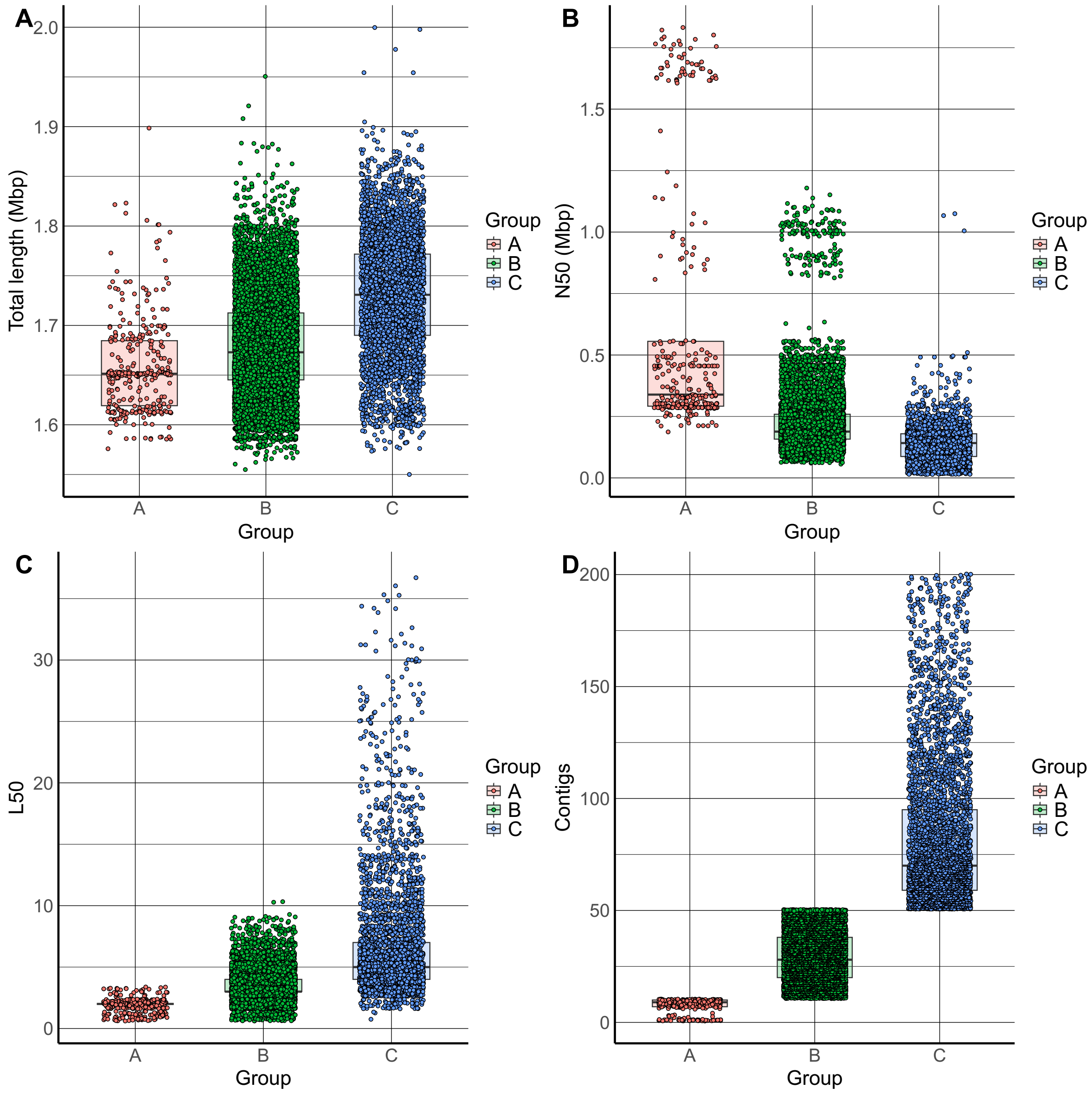

Figure S6. Analysis of *C. jejuni* genome assemblies using metadata categories. All analyses were performed utilising data contained within the *Campylobacter* PubMLST database. We have added to DataFile S1, the available summary statistics for all of the genome sequences, which includes the number and average lengths of contigs. In order to examine the degree to which our data may be influenced by genome sequence quality, the data was separated into three groupings relating to the number of contigs (A,B,C: 0-10, 11-50, 51-200 contigs respectively) as a reasonable measure of genome assembly quality. The number of contigs ranged from 1 to 200 while the genome size did not fall below 1.4 Mbp or exceed 2.1 Mbp, a criterion previously used by others [34]. We found that A, B and C had 313 ,11928 and 3889 isolates respectively. The median N50 value for each group (A=338,864 bp, B=188,359 bp and C=141,750 bp) was within the range previously used for *Campylobacter* genomes and the median L50 values were 2,3 and 5 contigs respectively [34,35,36].

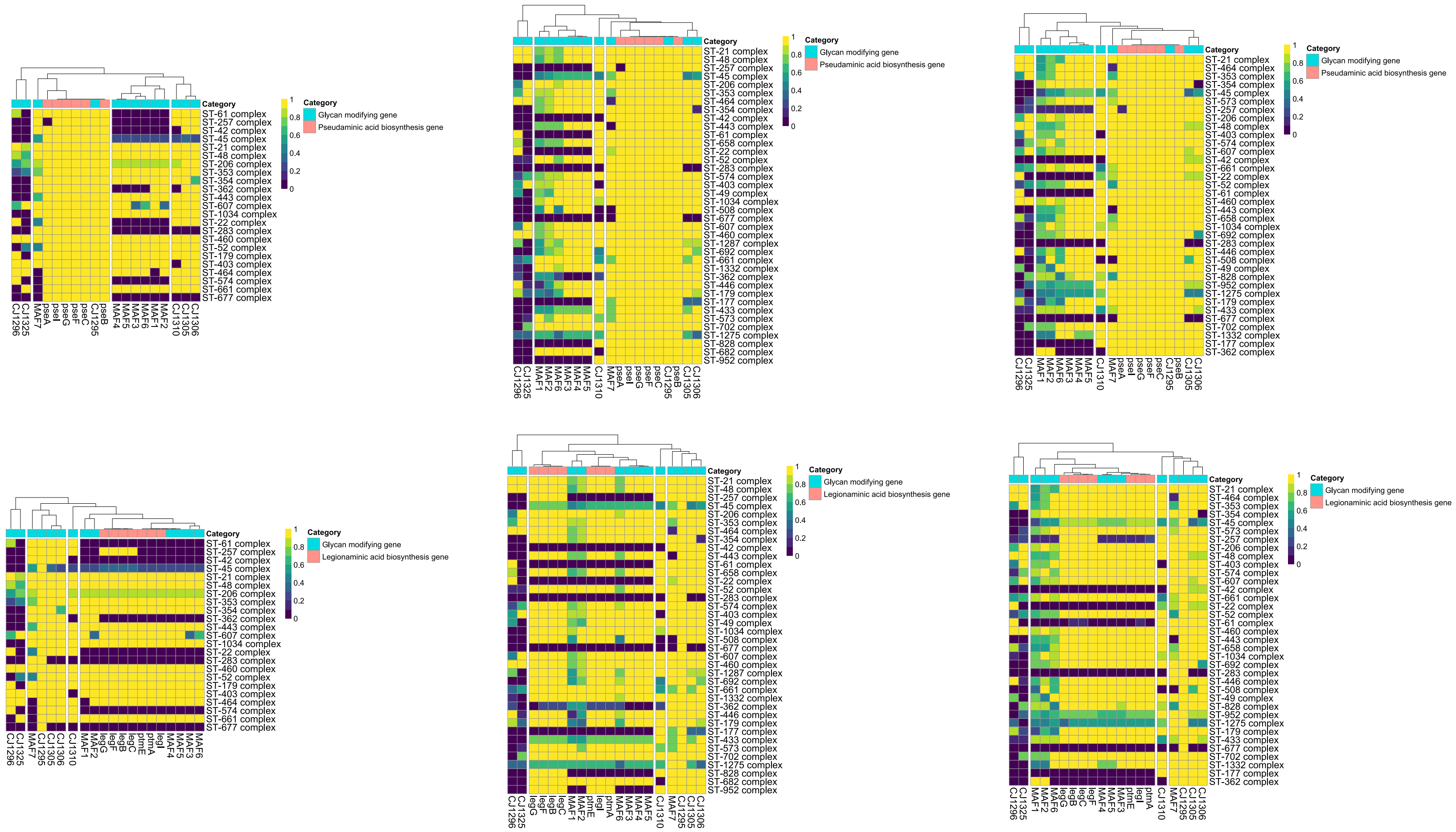

Figure S8. Heatmap analysis split according to genome assembly contig groups. Data from presence/absence association (Figure 2) was split into 3 categories based on the contig groups described in Figure S6 to observe any possible bias introduced by genome assembly quality. Image is arranged as a 2 by 3 frame. Rows (r) indicate Pseudaminic acid (r1) and legionaminic acid (r2) associations with glycan modifying genes. Columns (c) represent contig groups; A=c1, B=c2 and C=c3.

Table S1. Cloning primers.

Primer name	Sequence (5' -> 3')	Note
MAF3 L_Arm_F	aatactcaagctttgtttgaggtcg	Forward primer for amplification of left flanking region of *maf3* gene
MAF3 L_Arm_R	CAATAGGCCGCTCGAGgttgttttataagacttggtcctg	Reverse primer for amplification of left flanking region of *maf3* gene. Overhang region, homologous to kan_F primer, is underlined.
MAF3 R_Arm_F	CTGGATGAATTGTTTTAGTATCTAGcagaggagaaaaatatacactagc	Forward primer for amplification of right flanking region of *maf3* gene. Overhang region, homologous to kan_R, is underlined.
MAF3 R_Arm_R	cgcattcgttatgaaaataaatagc	Reverse primer for amplification of right flanking region of *maf3* gene.
BsmBI_pcmetK_MAF3_F	TTTAAATGAAAGGACTTTTTgtgagagaagagctttttt	For cloning of *maf3* gene into pcmetK plasmid. Overhang region, homologous to plasmid, is underlined.
BsmBI_L_ARM_MAF3_R	AGATAAATTAAAACGTCTCAttattttttgattatatcgtcaaaattcc	For cloning of maf3 gene into pcmetK plasmid. Overhang region, homologous to plasmid, is underlined.
FlaA_FlaB_DM_L_ARM_F	ccatgctaatttacacaatggagac	Forward primer for amplification of left flanking region of *flaA*/*flaB* genes.
Cj0046_iLOV_F	ctctctccgctagaaattaaatcc	For verification of ligation of inserts into pcmetK plasmid or pc46 plasmid
Cat_promoter_R	cgcctgagggattttatttattcagc	For verification of ligation of inserts into pcmetK plasmid or pc46 plasmid
